# Applications of Plasma Technologies in Recycling Processes

**DOI:** 10.3390/ma17071687

**Published:** 2024-04-07

**Authors:** Reinosuke Kusano, Yukihiro Kusano

**Affiliations:** 1 School of Physics and Astronomy, University of St Andrews, North Haugh, St Andrews KY16 9SS, UK; rk77@st-andrews.ac.uk; 2 Department of Marine Resources and Energy, Tokyo University of Marine Science and Technology, Tokyo 108-8477, Japan

**Keywords:** plasma, recycling, thermal plasmas, nonthermal plasmas, circular economy, plasma gasification, plasma reforming

## Abstract

Plasmas are reactive ionised gases, which enable the creation of unique reaction fields. This allows plasmas to be widely used for a variety of chemical processes for materials, recycling among others. Because of the increase in urgency to find more sustainable methods of waste management, plasmas have been enthusiastically applied to recycling processes. This review presents recent developments of plasma technologies for recycling linked to economical models of circular economy and waste management hierarchies, exemplifying the thermal decomposition of organic components or substances, the recovery of inorganic materials like metals, the treatment of paper, wind turbine waste, and electronic waste. It is discovered that thermal plasmas are most applicable to thermal processes, whereas nonthermal plasmas are often applied in different contexts which utilise their chemical selectivity. Most applications of plasmas in recycling are successful, but there is room for advancements in applications. Additionally, further perspectives are discussed.

## 1. Introduction

As billions of tons of industrial waste gradually gnaw at the health of the environment each year, the need to streamline and ameliorate existing recycling processes has become growingly more critical [1].

There are various types and kinds of waste emitted all over the world. They can be classified by the states of substances, uses, and materials. Taking Europe as an example, approximately 25.8 Mt of plastic wastes is generated every year, among which less than 30% is collected for recycling each year [2]. The majority of these are considered to be processed by primary recycling. Recycling processes for metals, which often use thermal plasmas, generally include sorting, squeezing/compacting, and melting in a furnace [3]. In 2018, 94 Mt of steel scrap was recycled. Other commonly recycled metals include aluminium (4.9 Mt), copper (1.6 Mt), lead, zinc, nickel, titanium, cobalt, chromium and specialty and precious metals (gold, silver, and platinum) [4].

Plasma, a reactive ionised gas known as the fourth state of matter [5], has proved to be very useful in the realm of waste treatment and recycling processes. Through the lens of recycling models, classifications, and economical models [6], this review discusses cutting-edge advancements of plasma technologies in recycling processes and evaluates their successes and shortcomings.

## 2. Classifications of Recycling

There are four main types of recycling: primary, secondary, tertiary, and quaternary recycling [7,8,9]. These are classified for plastics, but for the sake of analysis these definitions will be applied to general waste as well. There are different definitions in different literature, but the most commonly used definitions will be presented here.

Primary recycling is a closed-loop process, where the waste is only broken down to the level where its original chemical structure is not significantly modified [8,9]. It is a simple and cost-effective process. Recycling of polyethylene terephthalate (PET) bottles is a good example of primary recycling.

Secondary recycling involves downgrading the waste into less valuable materials [8,9]. Secondary recycling may be less desirable than primary, tertiary, or quaternary recycling, but is often otherwise inevitable. Secondary recycling is often employed to recycle inorganic substances, which are then mixed with concrete for reinforcement, for example.

Primary and secondary recycling are often collectively referred to as mechanical recycling [8,9]. However, this term may cause confusion or misunderstanding, since mechanical recycling generally involves not only mechanical processes but also heating and mixing (which may include melting), as well as chemical reactions. Hence, since the term is not rigorously defined in literature, this expression is not used in the present review.

Tertiary recycling refers to chemical recycling and thermal recycling. Thermal recycling, in which the waste is broken down to a molecular level [9] is different from combustion. Quaternary recycling involves the thermal combustion of the waste and aims to use the energy generated from this process [8,9]. Tertiary or quaternary recycling is often employed for wastes which are difficult to be recycled in a simple manner, often due to their complex structures or contents.

**Figure 1:**
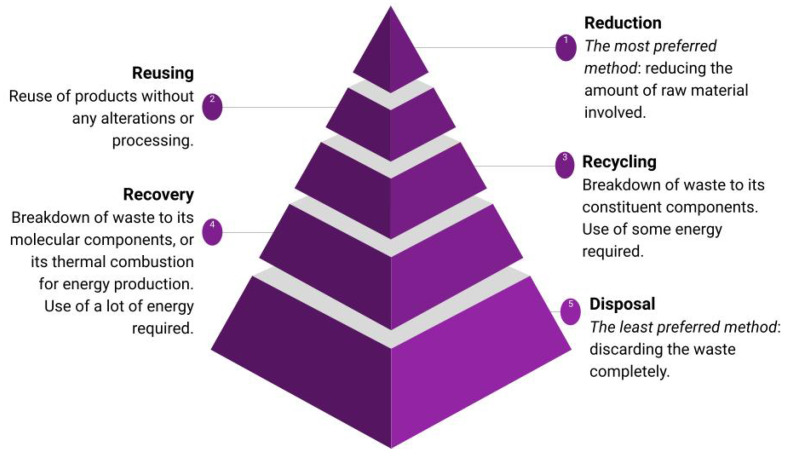
A diagram of the waste management hierarchy model.

Different types of waste disposal models are also explored. First, there exists a model known as the waste management hierarchy model, as shown in Figure 1. This model ranks the desirability of different types of waste management, raking the most desirable at the top and the least desirable at the bottom. Taking plastic as an example and starting from the most preferred method:

- Reduction (reducing the amount of plastic material produced in the first place);
- Reuse (reusing the used plastic);
- Recycling (breaking down the used plastic to its constituent components, corresponding to primary recycling);
- Recovery (breaking down the used plastic to its molecular components or using them for energy production, corresponding to tertiary recycling, as well as the generation of energy from quaternary recycling);
- Disposal (throwing away the used plastic).

Essentially, the purpose of this model is not too dissimilar to the circular economy model, which is discussed later: the fundamental concept is to minimise the amount of processing i.e., the amount of change required to make the waste reusable again, at least to some capacity.

What is recycled and what is discarded as waste depends on what kind of waste is being processed. For example, with plastics, one may keep the organic components as syngas (a gas mixture of H_2_ and CO), while in the recovery of metal waste one often discards organic components.

**Figure 2:**
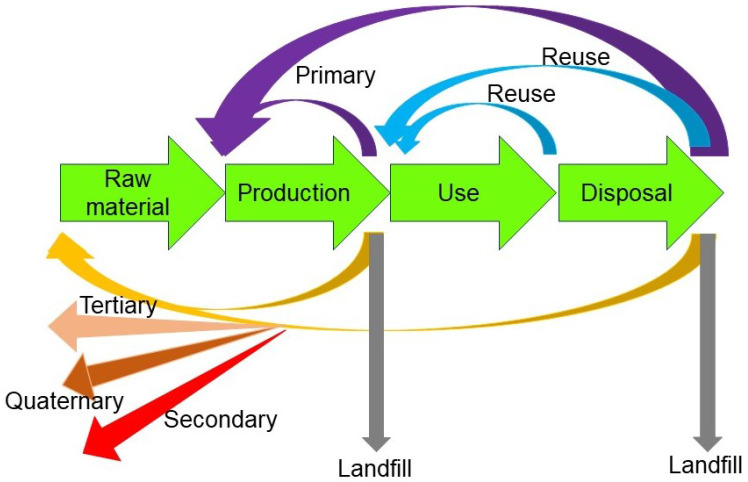
A diagram of the circular economy model.

Additionally, a circular economy, as depicted in Figure 2, is considered. A circular economy is a circular model of production and consumption, aiming at extending the life cycle of products as much as possible [10]. Specifically, waste should be reduced to a minimum, while end-of-life products should be kept within the economy by recycling to create further value. The concept of circular economy helps increase the amount of reused or recycled plastic in the overall cycle [11], to ensure that one prevents the needless discarding of plastics as much as possible. This is achieved by creating closed loops within the waste management process, and “looping back” with a given piece of waste to a given point on the progression, such that as much of the waste goes through the cycle as possible. As can be seen, secondary, tertiary, and quaternary recycling do not form a closed circular loop.

Something to consider with regards to a circular economy is that smaller loop sizes correspond to higher efficiencies. This is because as the size of the loop increases, this inevitably leads to an increase in the energy used in the processes needed to break down the plastic to its constituent components.

In this model, extra materials or defective materials produced during the manufacturing process may also be taken into consideration. This is important; to start with raw materials that are initially unused makes reprocessing easier. Additionally, defective materials found during manufacturing can be very easily processed for recycling, since their components are already known by the manufacturer. Environmentally speaking, this results in a net positive; while the production of waste is inevitable and new materials have to be added to the system constantly, using materials of known origin boosts their reusability. With regards to the classifications of different recyclable products, it is also worth mentioning that the circular economy is applicable to organic and inorganic substances.

The four-level classification (primary, secondary, tertiary, and quaternary), waste management hierarchy, and circular economy are different in a few ways. The four-level classification groups different types of recycling in terms of the process utilised, while the waste management hierarchy groups recycling processes in terms of their environmental friendliness. On the other hand, the circular economy attempts to describe the dynamic flow of recycling processes. Therefore, the processes described in the four-level classification and waste management hierarchy appear in the circular economy model. Combining these different recycling models helps classify a given recycling process in terms of effectiveness, accessibility, and need.

One major drawback of the circular economy model is that there are many cases where a loop is not actually formed in the recycling process. Therefore, it must be understood that the circular economy model with a closed loop is an idealistic one.

## 3. An Overview of Plasma

Plasma is often known as the fourth state of matter that contains electrons, ions, and radicals [5,12], which in nature can be seen in, e.g., stellar structures [13,14] and the northern lights [15].

In the waste management community among others, plasmas are generally classified into thermal and nonthermal plasmas. Thermal plasmas, also called high-temperature plasmas, have high energy densities and are very productive in terms of processing speed. In these types of plasmas, the gas temperature and the electron temperature are high, and the plasma is at an equilibrium state or is almost at an equilibrium state. Additionally, the gas molecules inside a thermal plasma are fully or mostly ionised. Meanwhile, nonthermal plasmas, otherwise known as low-temperature plasmas, cold plasmas, or non-equilibrium plasmas [16], are only weakly or partially ionised. An important characteristic of the nonthermal plasma is that the electron temperature is much higher than those of ions and neutrals, and in fact, in some cases can be even higher than the electron temperature in a thermal plasma. The electron temperature in a nonthermal plasma can be, for example, 10^4^–10^5^ K, corresponding to ca. 1–10 eV, while the temperatures of the ions and neutrals can be as low as room temperature, or often up to several hundred K [17].

In the following two subsections, the different advantages and disadvantages of thermal and nonthermal plasmas are discussed in detail. In short, thermal plasmas are suitable for high-speed processes, while nonthermal plasmas are favourably used for selective chemical processes [18].

### 3.1. Thermal Plasmas

**Figure 3:**
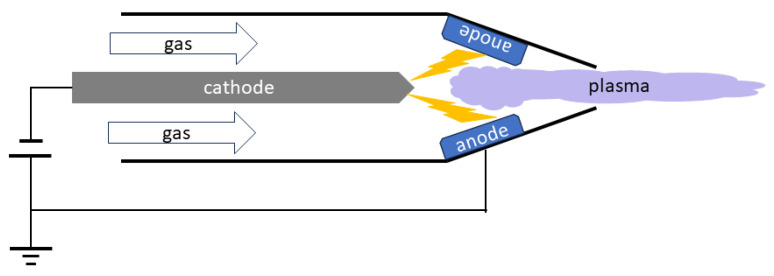
A configuration for a direct current (DC) thermal plasma torch.

**Figure 4:**
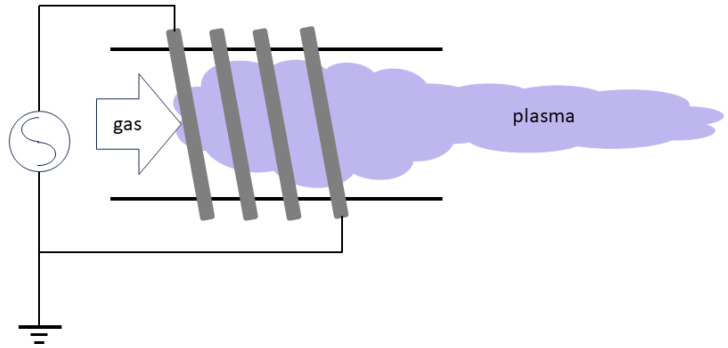
A configuration for a radiofrequency (RF) thermal plasma torch.

**Figure 5:**
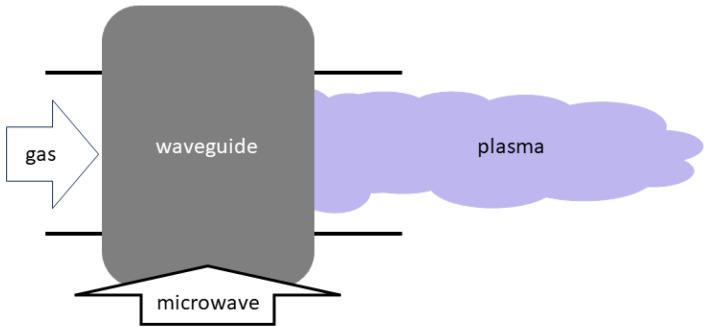
A configuration for a microwave (MW) thermal plasma torch.

Thermal plasmas can be operated with very high power, and the reaction process is also very fast, since the voltage required to sustain them is very low. Plasma torches using thermal plasmas are commonly used for waste handling processes. The main disadvantage of thermal plasma processes is the fact that such a process is essentially a thermal process, and therefore is has low chemical selectivity; if given the opportunity to do so, it will react indiscriminately with all target particles. Different configurations for the generation of thermal plasmas are shown in Figure 3, Figure 4 and Figure 5. Figure 3 is the design for a direct current (DC) plasma torch, Figure 4 is the design for an inductively coupled radiofrequency (RF) plasma torch, and Figure 5 is the design for a microwave (MW) plasma torch. In all cases, thermal plasmas are generated by introducing electrical energy and are extended by a gas flow. Waste is then exposed to the extended plasma.

Spark plasma is also a thermal plasma that is similar to an arc plasma. These technical terms are often interchangeably used. However, arcs are continuous while sparks are momentary. Spark plasma is generated by applying a pulsed electric field between electrodes.

An overview of the types of plasma that can be used in recycling processes are given below in Table 1.

### 3.2. Nonthermal Plasmas

The main advantage of nonthermal plasma is its selectivity due to its high electron temperature [5]. This means that the desired reaction paths for a given process can be easily designed using this type of plasma. Since the gas temperature is low, nonthermal plasmas typically do not affect the bulk properties of solids or liquids but interact only with their surfaces [5,12,28,29]. Therefore, nonthermal plasmas are preferably applied to surface [30,31,32,33,34,35,36,37,38,39,40,41,42,43,44,45,46,47,48,49] or gas processing [50,51,52,53,54], and their comparative safety to thermal plasmas also make them applicable to medical and oncological processes [55,56,57,58,59,60]. In fact, recent progress in plasma treatment of cancer may be considered a worthwhile addition to more popularly addressed cancer treatments like single-cell RNA sequencing [61].

The main shortcoming, however, is that nonthermal plasmas are unable to take a high energy density; hence, they are not as productive as thermal plasmas in terms of processing speed. Additionally, nonthermal plasmas are often operated at low gas pressure at which it is easy to generate such types of plasmas; the downside to this is that low gas pressures require expensive vacuum systems. Although plasma at atmospheric pressure is desirable, if one attempts to increase the gas pressure, the temperature of the gas and ions in the plasma can easily increase. This is because the increase in gas pressure leads to an increase in the frequency of inelastic collisions between the electrons and the other particles in the plasma. In order to sustain the plasma, the input power has to be increased. Therefore, at higher pressures, the heating of the gas is accelerated and the nonthermal plasma evolves to a thermal plasma [12] at a very high likelihood, although there are several techniques with which nonthermal plasma can be stably operated at a high gas pressure [5,12,28,29,62].

Nonthermal plasmas generally used for processing materials include low-pressure plasmas and atmospheric-pressure plasmas. Low-pressure plasmas are generally operated by DC, RF, or MW. Atmospheric-pressure plasmas, which do not require an expensive vacuum system, is preferred for recycling processes. Atmospheric-pressure plasmas may be generated using DC (which include pulsed operation), high frequencies up to but less than a megahertz, RF, or MW [62].

Different types of atmospheric-pressure plasmas have been developed, which include corona discharge, dielectric barrier discharge (DBD), cold plasma torches, and gliding arcs [28,62].

**Figure 6:**
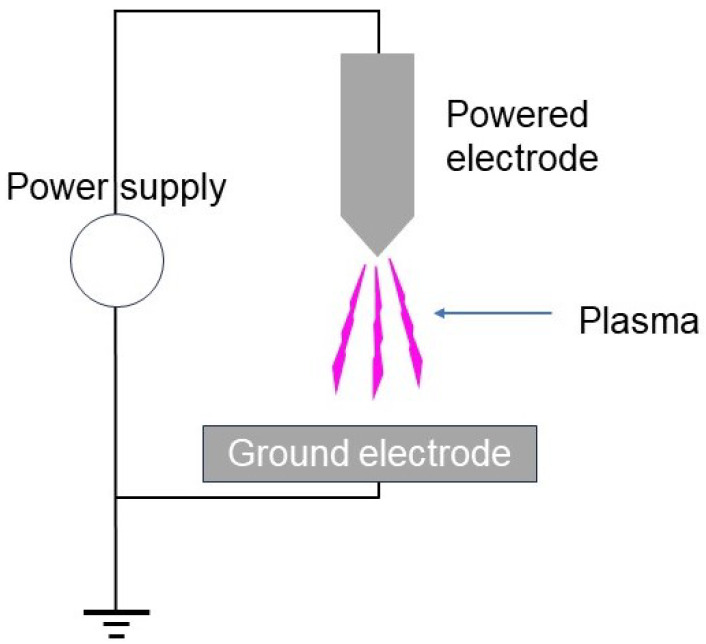
A configuration of corona discharge.

A corona discharge is generated in a significantly non-uniform electric field, usually appearing in the vicinity of sharp edges of electrodes, as can be seen in Figure 6. The discharge current must be limited to avoid the plasma becoming an arc. This is done by applying pulsed high voltages, resulting in an expensive power supply and low speed processing.

**Figure 7:**
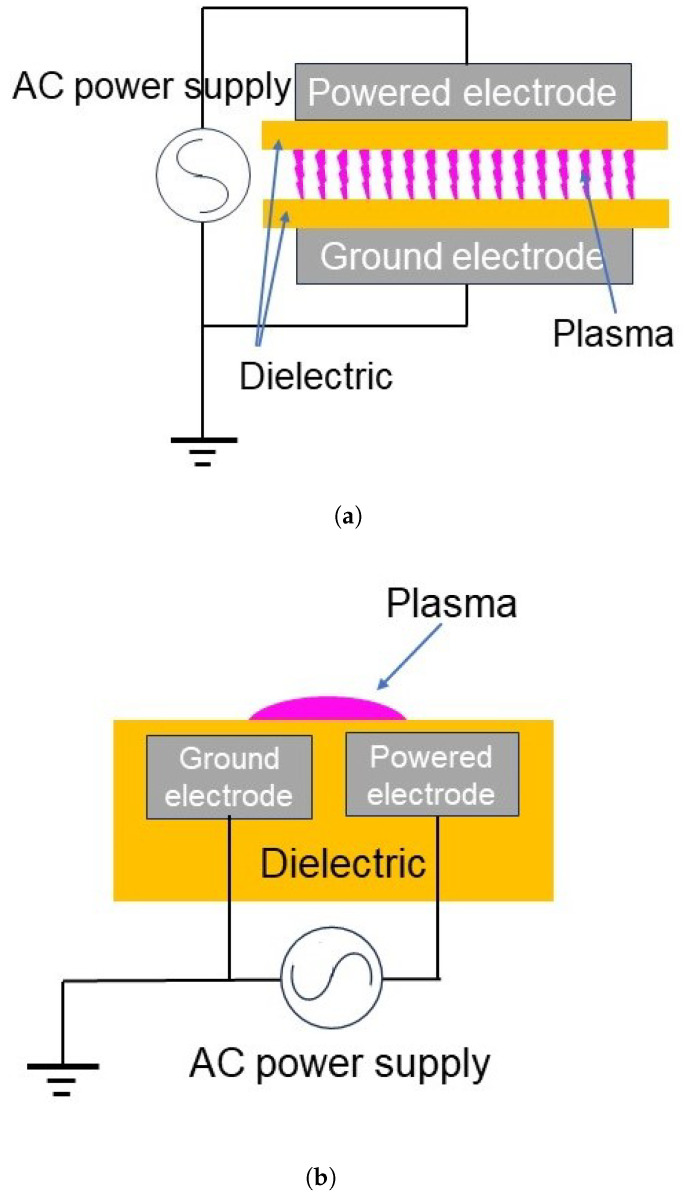
Configurations of DBD plasmas. (**a**) Volume discharge. (**b**) Surface discharge.

DBD is generated between electrodes by applying an alternating current (AC) voltage. At least one dielectric insulator is inserted between the electrodes to block DC. DBD is the most widely used atmospheric-pressure plasma in materials processing [31,32,33,34,35,36,37,38,39,40,41,42,43,44,45,46,47,48,49,51,63]. Two configuration of DBDs are known, as illustrated in Figure 7. As in Figure 7a, a volume discharge is generated at the gap between the electrodes. The gap is typically limited to several millimetres. A surface discharge is generated at a dielectric material surface with an electrode on each side, or with all electrodes embedded in the dielectric material, like in Figure 7b. Alternatively, two electrodes are attached on one side of the dielectric material, while the surface discharge is generated on its reverse side. In this case, a surface discharge is achieved by feeding a specific gas such as helium or argon to the reverse side. The frequency required to operate a DBD is typically between 500 Hz and 500 kHz, but DBDs at 50 Hz are also reported [42]. As the frequency is lowered, capacitive impedance increases at a fixed gap, and at a fixed applied voltage the power is lowered. Therefore, in general, a higher frequency is preferred for high-speed processing.

**Figure 8:**
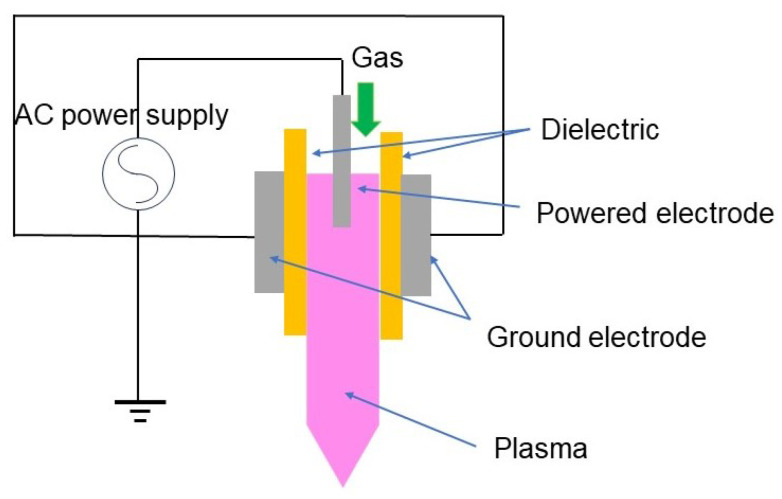
A configuration of a cold plasma torch.

In most cases, DBDs consist of non-uniform filamentary discharges, and subsequently, the plasma treatment is not uniform. However, it is possible to generate uniform DBDs, and such a discharge is called a glow discharge. It is reported that a filamentary discharge can be avoided by using a noble gas such as argon or helium [28,29]. It may also be avoided using short pulses or by operating DBDs using RF. When RF plasmas are used, the dielectric is unnecessary. However, the operation of DBDs using RF requires an impedance-matching network to deliver RF energy to the plasma, usually limiting high voltage outputs. Due to the limitation of the high-voltage application, the electrode gap must be small enough for the stable operation of the discharge. In this respect, atmospheric-pressure RF plasma is rather preferably developed for cold plasma torches (depicted in Figure 8), which do not require the insertion of a specimen between the electrodes [64,65].

DBD and other plasmas, which are typically capacitively coupled plasmas, can be extended by a gas flow of argon or helium into an ambient air to realise cold plasma torches. They are typically weakly ionised [12,28]. A cold plasma torch can be operated at a high frequency (kHz) or RF in a DBD [12], or without covering electrodes with the dielectrics [12].

**Figure 9:**
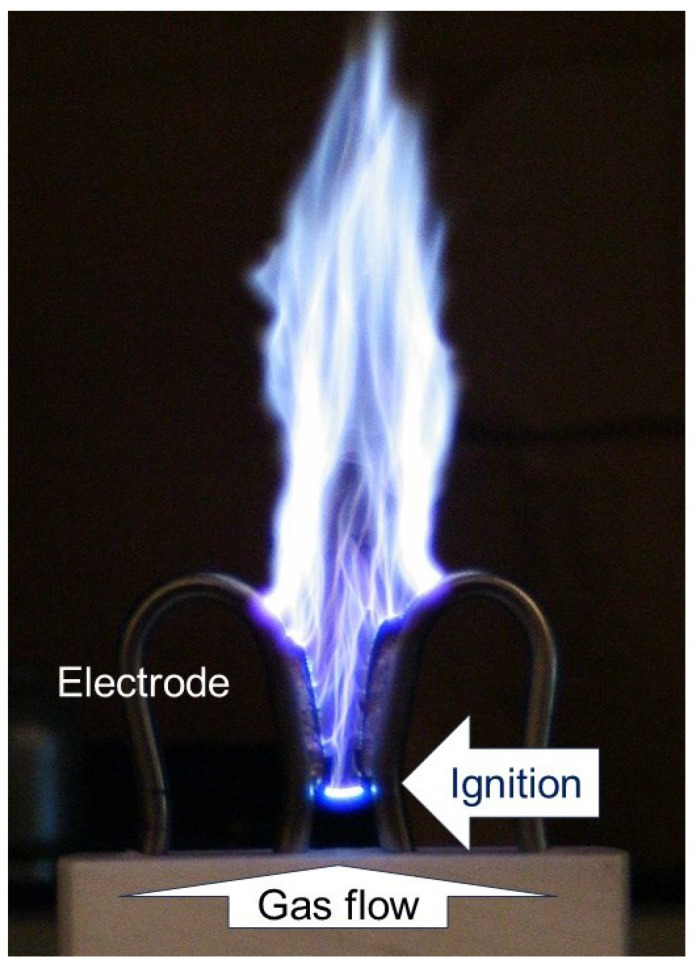
A photograph of a gliding arc.

It is desirable that atmospheric-pressure plasma processing demonstrates a hybrid condition, which simultaneously achieves high chemical selectivity and efficient productivity [12,34,62]. In most cases, neither thermal nor nonthermal plasmas are able to provide them simultaneously. However, a gliding arc can achieve such a hybrid condition [5,12,16,34,62]. A gliding arc, as seen in Figure 9, is generated between diverging electrodes, and it is extended and quenched by a gas flow [12,16,27,63,66,67,68,69,70,71,72,73,74,75,76,77]. Atmospheric-pressure MW plasmas can also be generated and extended in a similar way. When the gas flow is fast enough to quench the plasma, its properties are similar to those of the gliding arcs [12]. However, using MW is argued to be less economical [18,62]. An industrially available open-air technology called technology “Plasmatreat^®^” is also a plasma generated as an arc that is extended and quenched by a gas flow, and its performance is similar to general gliding arcs.

### 3.3. Terminologies

There are inconsistencies in the literature regarding the classification of plasmas. There are, for example, publications claiming that high-temperature plasmas are only seen in stellar structures and fusion reactors and that group both thermal and nonthermal plasmas as low-temperature plasmas [78]. However, this claim requires reconsideration on a few fronts and may be cause for confusion.

Firstly, the claim is semantically undesirable. If thermal plasmas were low-temperature plasmas, they should not be called thermal plasmas in the first place.

Secondly, this claim is fallacious where astrophysical phenomena are considered. There are stellar prominences known as slingshot prominences, which are effectively coronal plasma condensations on rapidly rotating stars [79]. For the coronae of stars with temperatures at around 10^6^ to 10^7^ K, the temperatures of slingshot prominences are set at around 8500 K [79,80]. It is true that the coronae are at very high temperatures, but the temperatures of the prominences themselves can be easily achieved using DC thermal plasma torches, as their maximum temperature is somewhere around 20,000 K [81]. This is to say, according to the aforementioned claim, a slingshot prominence at 8500 K may be considered a high-temperature plasma, while simultaneously a DC-generated thermal plasma at 20,000 K is considered a low-temperature plasma. These are incompatible, and hence claiming that thermal and nonthermal plasmas are both low temperature is inappropriate. If high-temperature plasmas correspond to an environment of fusion reactions, it is suggested that this kind of plasma is called a fusion plasma.

Another cause for possible confusion regarding plasmas is the fact that plasma arcs and plasma torches have been differentiated, and plasma arcs have been categorised into transferred arcs and non-transferred arcs [78]. However, it is known that transferred and non-transferred arcs are essentially plasma torches [81]. In fact, the plasma torch uses an arc which is extended by a gas flow. Furthermore, the technical term “plasma arc” should be avoided, since an “arc” is an arc plasma. Hence, the categorisation and differentiation of plasmas into plasma arcs and plasma torches is unnecessary.

The term “plasma arc” originated from “plasma arc welding”, which is essentially the use of plasma torches. It is speculated that “plasma arc” just isolated itself from this term, as it sounds attractive on its own. However, in the realm of plasma physics it is desirable to append the word “plasma” as a suffix rather than a prefix, such as in, e.g., arc plasma, DBD plasma, etc.

To avoid these confusions in the recent literature, the present review takes the following definitions for plasma:

- Thermal plasmas refer to high-temperature plasmas, which are at an equilibrium state or almost at an equilibrium state;
- Nonthermal plasmas refer to low-temperature plasmas which are at a non-equilibrium state, or more specifically, whose electron temperature is much higher than the temperature of the gas.

These definitions are not unique—they have already been established in other literature reviews [82]. With this background, different types of plasma processes in recycling are reviewed, in conjunction with different types of economic models.

## 4. Thermal Plasmas in Recycling Processes

Thermal plasmas are most extensively used in recycling. They include plasma torches and spark plasmas. In both cases, plasma is used to effectively heat up solid waste. Plasma torches are commonly employed in plasma gasification (see Section 4.1) and metal recovery (see Section 4.2), while spark plasmas are used to sinter ceramics (see Section 4.3). Plasma torches are further classified into three different types in terms of their operation frequencies. They are DC, RF, and MW plasma torches as shown in Figure 3, Figure 4 and Figure 5. Table 2 summarises the differences in these plasma torches.

These are essentially the same with some minor differences, which make them suitable for specific situations. Detailed descriptions are presented in Section 4.1 and Section 4.2.

### 4.1. Plasma Gasification

A commonly used recycling process that utilises thermal plasmas is the thermal gasification process. Plasma gasification produces a syngas (H_2_, CO) which can be used as a fuel. The process also produces raw materials from which valuable resources, such as hydrocarbons or ammonia, may be synthesised. It is evident that this process corresponds to the recovery process in Figure 1, and therefore this is a tertiary recovery process in Figure 2.

The plasma gasification process is a leading method for solid-waste treatment [87,88,89,90,91,92,93,94,95,96,97,98,99,100,101,102,103,104,105,106,107,108,109,110,111,112,113,114,115,116,117,118,119,120,121,122,123,124,125,126,127,128,129,130,131,132,133] and has been reviewed quite extensively over the past several years [82,94,95,96,99,134,135]. This process, which usually involves thermal plasmas, is considered very effective and environmentally friendly [109,110,134,136,137,138,139,140,141,142,143,144,145,146]. One important thing to note about the plasma gasification process is that it is a purely thermal process.

While there technically do exist gasification processes that do not utilise plasmas, many cases where the term “gasification” is used implicitly refer to plasma gasification.

Done at high temperatures, plasma gasification entails the thermal decomposition of waste. In the case of inorganic compounds, these are usually oxidised and turned into slag, which can either be disposed of [147] or reused in materials like concrete [148], while the organic compounds are turned into a syngas which has a double merit; not only is it a fuel, but it is also cleaner than its more popular counterparts [87].

Syngas is environmentally friendlier than what is produced in gasification processes that do not involve plasma, since hydrocarbons are produced for such processes. The burning of hydrocarbons results in the production of CO_2_, whereas burning the hydrogen gas in syngas from plasma gasification only produces H_2_O [107]. In the past, CO was also used as a fuel [149], but it has recently been avoided due to its toxicity [150]. It is also worth noting that while the components in the syngas may be used as a clean fuel, they can also be used to make, e.g., ammonia (NH_3_), methane (CH_4_), methanol (CH_3_OH), formaldehyde (HCHO), or formic acid (HCOOH).

**Figure 10:**
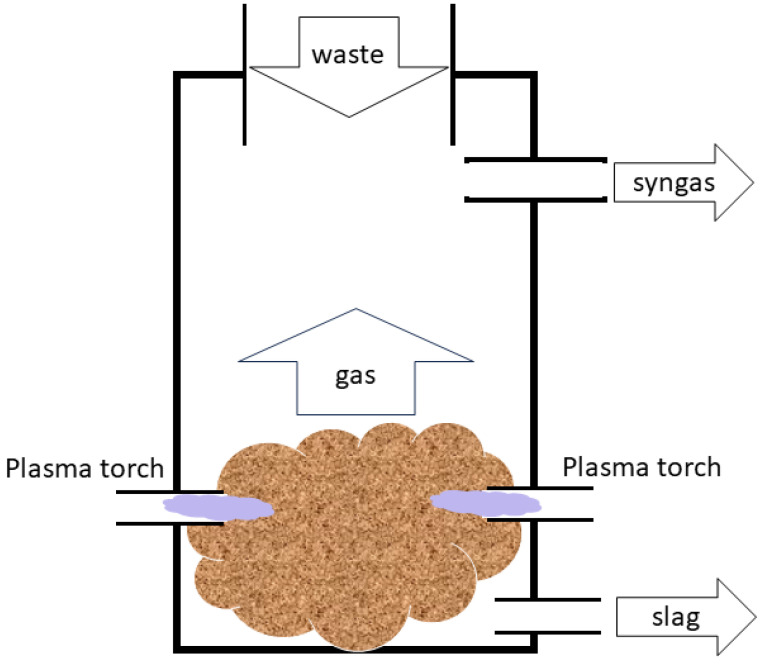
A diagram of the plasma gasification process.

A simplified schematic diagram of a typical plasma gasification process is shown in Figure 10.

Plasma gasification using thermal plasma involves a plasma torch, in which an electric arc is generated and extended by a gas flow to heat the waste. The gas used to extend the electric arc is often air, or oxygen-rich air. Sometimes, argon (Ar) is introduced as well so that the ignition becomes easier at a lower voltage [12].

As for the plasma itself, its temperature can generally reach around 13,000 °C but touches the waste in question at a much lower temperature, between 2700 to 4500 °C, which is more than enough to decompose the target waste [151].

In recent publications, one can often find the terms “plasma pyrolysis” and “plasma gasification”. Technically speaking, plasma pyrolysis is thermal decomposition of target waste in “the near absence of oxygen” [152], while the presence of oxygen is encouraged in plasma gasification. However, these two terms are often used as synonyms [81,82,135,153], and hence they are used synonymously in this review as well.

As illustrated in Figure 3, Figure 4 and Figure 5, there are three main types of plasma torches that are used for plasma gasification: DC plasma torches, RF plasma torches, and MW plasma torches [81].

Due to the availability of large-scale power supplies, DC plasmas are capable of generating a power output in the range of megawatts. Their main shortcoming is that since the temperature of their arc is so high, the electrodes start to erode. Hence, impurities are generated in the arc, and this affects the quality of the products which are processed by DC plasmas [81].

RF plasmas can be generated in two ways: it can either be generated as an inductively coupled plasma (ICP), or as a capacitively coupled plasma (CCP). In the ICP, the time-varying magnetic field induces an electric field, whereas in the CCP, the electric field is directly oscillated. The electron density can be higher in the ICP, whereas with the CCP, a DC self-bias can be generated with an appropriate impedance-matching network, such that the ions in the plasma are accelerated toward the powered electrode. This can be used for physical etching of the target material. One must consider here that in gasification, a rise in temperature is necessary; therefore, the ICP is more suitable in this case [154]. If it is an ICP, RF plasmas are operated without the use of an electrode, and hence do not suffer the disadvantage of generating impurities—the caveat being that they are less efficient compared to DC plasma, generating only a few hundred kilowatts [81,135]. Additionally, there are some issues with impedance matching [81,135].

MW plasmas can have a higher plasma density than RF plasmas, and their plasma zone can also be much larger [78]. Their other advantages include that they are highly effective and only require a “simple and robust” design for their reactor [82], while also being a low-voltage operation. Additionally, they do not have any issues with impedance matching, and they do not require electrodes either [135]. These are the reasons why some researchers in the field consider MW plasmas to be the most superior type of thermal plasmas [135]. However, MW plasmas do come with their own set of shortcomings. First, MW plasmas generate the least amount of power out of the three types; they are generally able to generate up to only 10 kW of power [81]. Furthermore, while they require the least amount of power in sustaining the arc, the initial ignition of the arc requires a comparatively high voltage [135] and thereby a separate ignition system, while neither DC nor RF plasma requires this.

It is interesting to see just how many areas of waste management plasma gasification is used in: the treatment of medical waste [141,155,156] and municipal solid waste (MSW) [142,157], as well as deriving fuel from enhanced landfill mining (ELM) [158] and from the conversion of coal [159] and biomass [160,161], as well as plenty more.

However, plasma gasification proves to be disadvantageous through the lens of economic viability [113,162]. With regards to this, it is highly debatable whether plasma gasification is necessarily the most efficient method of waste decomposition. As will be discussed in Section 5.1, although low-temperature plasma (i.e., nonthermal plasma) may also be used to gasify waste [91,96,118], the plasma gasification process primarily involves thermal plasma which, as stated previously, inevitably involves an extremely high processing temperature [96,118]. Additionally, it is not always the case that the waste product needs to be decomposed into its molecular constituents, at least not to the extent that plasma gasification allows. Here, one must take the waste management hierarchy model and the circular economy model into consideration. If waste was decomposed to its molecular components every time, the process is not as energy-efficient or as cost-effective as reusing a less-decomposed part of the original material.

In terms of the waste management hierarchy model [163] in Figure 1, plasma gasification corresponds to the recovery step, which is far from ideal. In other words, purely economically speaking, plasma gasification is not the most ideal method of recycling using plasmas. However, it is worth noting that the major advantage of plasma gasification renders the sorting of waste into specific categories unnecessary or at least reduces its need, since the waste is decomposed into its molecular constituents anyway.

Currently, a motivating factor of plasma gasification research seems to be to maintain the high gas temperature while decreasing the input power [135], as the high input power is a major setback of the thermal plasma. However, since this type of plasma is inherently characterised by a high input power and a high pressure, this is not an optimal solution. One can only hope that the theoretical framework of plasma gasification is further developed to fix its setbacks [82], and that the development of numerical models [142,156,157,161,164,165,166,167,168,169,170] will optimise the efficiency and effectiveness of this process in the future. For example, it may be advantageous for future numerical methods to simulate a situation in which a temperature limit is set for the plasma discharge, such that the target material is selectively thermally decomposed. For instance, selecting an appropriate temperature window may facilitate selectivity [142,156,157,161,164,165,166,167,168,169,170].

### 4.2. Recovery of Metals

Plasma processes are also commonly used in the recovery of inorganic materials. For example, as mentioned earlier, slag can be recovered in the gasification process for use in construction.

Thermal plasma may also be used in the recovery of metals from waste. Most of these cases involve thermal plasma torches. The recovery of metals also corresponds to the recovery process in Figure 1 and therefore corresponds to tertiary recycling in Figure 2. Pyrometallurgy and hydrometallurgy are commonly used techniques for recycling metal. Pyrometallurgy is a thermal recycling technique that extracts metals from electronic waste, often using thermal plasmas. In other words, if pyrometallurgy is employed, a thermal plasma process is often involved. Hydrometallurgy is a wet process using liquids. It does not require high temperatures but emits significant amounts of waste liquids.

For example, the recovery of metals from household uses, industrial applications such as steel, aluminium, and copper, and electronic waste is one of the most popular applications of thermal plasma [3,171,172,173,174,175,176,177], and it is commonly performed with secondary batteries and waste circuit boards. However, the recycling of secondary batteries is complicated, so it is discussed further in Section 5.3. For waste circuit boards, a plasma torch is used to ensure that the organic molecule macrostructures in the circuit board are destroyed to generate a syngas (plasma gasification), and the bonds connecting the metals and fibreglass boards are also broken efficiently [172]. However, the main purpose of this process is not in the generation of combustible gases but rather the recovery of metals from the waste. Indeed, the remaining metals exist in a mixture [175,177]. This mixture of metals can then promptly be coupled with acid leaching and a depolariser to yield desirable metals like copper, aluminium, and iron [177]. DC plasma torches are most often used for this process, as the high temperature improves the separation of the slag metal [175]. In all cases, thermal plasma torches are used, similarly to the plasma gasification process.

There are of course other applications of plasmas in the recovery of metals from different types of waste. For example, another application of plasmas in this area is the recovery of pig iron from red mud [178]. Red mud is a hazardous residue that is separated from sodium aluminates during the Bayer process [178,179]. A DC plasma torch is used for this process [178].

Similarly, electroplating sludges usually come from electroplating plants and consist of a large quantity of heavy metals [180,181]. While metals like chromium and nickel among others can be obtained from this waste using vitrification [180], in-flight treatment of the waste with a DC plasma torch has also been used to obtain these same metals [182].

For galvanic sludge, a byproduct of industrial metal surface treatment, a DC plasma torch is used for the decomposition [1,175].

Lastly, aluminium dross (or slag), which is considered an odorous hazardous waste [1,175,183], may be treated using plasmas. This may achieved by using a plasma torch to recover aluminium at a very high efficiency [175,184], or by applying an MW plasma torch to produce calcinated alumina [183].

For a comprehensive review of these processes, consult the review by Changming [175].

It is important to note, however, that some also consider the decomposition of zircon to fall under this category [175]. This is not the case. It is indeed true that zircon refractories may be thermally decomposed using plasmas [185,186], but these processes do not belong in this waste decomposition category as zircon is not waste. Zircon is the source for zirconia, a mineral with many uses [187], and additionally has been considered as one of the leading hosts for radioactive waste [188,189]. Hence, zircon is not waste, and should not be considered as such.

### 4.3. Spark Plasma Sintering

Spark plasma is used to sinter ceramics. The process is called spark plasma sintering, which is also called plasma pressure compaction or pulsed electric current sintering, and it is additionally referred to as a field-assisted sintering technique [190,191,192,193,194,195,196,197,198,199,200,201,202,203,204]. Spark plasma sintering is a pressure-assisted sintering process, in which a material is subjected to a simultaneous uniaxial pressure and high-intensity pulsed current induced by a spark. It is similar to hot pressing, but in spark plasma sintering, thermal energy is directly provided to a material by a current flow in a plasma. The original concept was proposed in 1912 [205], but its practical use started in the 1980s after the first laboratory-scale systems were developed [206]. The development of commercial spark-plasma-sintering systems induced a wide range of use for sintering.

Spark plasma sintering is also applied to waste processes. Spark plasma sintering is proposed as a solid-state recycling technique [207], exemplifying the consolidation of aluminium alloy scraps [208]. Calcareous waste concrete powder is processed by spark plasma sintering [209], improving the flexural strength and modulus of the sintered bodies. It is reported that rice straw agricultural waste can be processed by spark plasma sintering to produce aluminosilicate matrices for caesium immobilization [210]. Calcium silicate matrices are manufactured from boric acid production waste using spark plasma sintering for removing and immobilizing ^60^Co [211].

## 5. Nonthermal Plasmas in Recycling Processes

Nonthermal plasmas are characterised by their high electron temperatures and low gas temperatures, as listed in Table 1. The electron temperature is high enough to affect the chemical bonding of molecules, while the gas temperature may be as low as room temperature. They are therefore suitable for surface chemical processes and gas-phase chemical processes. Gas-phase reactions in nonthermal plasmas typically involve several hundred reactions [212]. On the other hand, since the gas temperature is low, nonthermal plasmas do not exhibit optimal performance if a thermal process is desired for solids or liquids.

Nonthermal plasmas may also be applied to waste recycling processes. A number of their applications are listed below, and are discussed in detail in the following subsections:

- Plasma reforming;
- Wind turbine blade recycling;
- Battery recycling;
- Paper recycling.

In short, plasma reforming is an example of gas-phase reactions. In wind turbine blade recycling, there has been an attempt to use nonthermal plasma for a moderate thermal process with less success. Recycling processes for lithium-ion batteries and papers employ plasma surface modifications.

### 5.1. Plasma Reforming

Plasma reforming is a very broad term that entails the production of usable fuels and raw materials. Reforming is not only used for waste handling, but also the conversion of organic compounds like methane or other hydrocarbons and alcohols to hydrogen, syngas, or valuable organic molecules [213,214,215].

Plasma reforming using nonthermal plasmas, at least in the literature, considers only gas-phase reactions, since bulk properties of solids or liquids are not significantly affected by the nonthermal plasma. Plasma reforming encompasses both (nonthermal) plasma gasification and plasma liquefaction; these processes are possible using nonthermal plasmas, by tuning the selectivity of reactions. The major difference between nonthermal plasma gasification and plasma liquefaction is the state of the main product at room temperature, whether it be gas or liquid; otherwise, they are essentially the same. The term “plasma gasification” is mostly used for the thermal plasma process. To avoid confusion, this review collectively refers to both nonthermal plasma gasification and nonthermal plasma liquefaction as plasma reforming.

Nonthermal plasmas are often used in the production of hydrogen from waste plastics [216,217,218,219], or for the conversion of methane [220,221,222,223] or other organic materials to hydrocarbons [224,225]. A high selectivity is needed for such a process, and therefore, nonthermal plasmas are desirable.

It is interesting to see that plasma reforming is often used for the treatment of organic materials [224,225,226,227,228,229]. Namely, gasification or liquefaction via nonthermal plasmas is often used in combination with biomass [230,231]. For example, this process is used to combine biorefinery with the chemical recycling of plastics [232], to upgrade biomass vapours so that biofuels may be produced [230], or for the refinery of biomass by removing tar from it [231]. The last process particularly benefits from the plasma reforming process, as the level of tar produced from this process is less than 0.1% of the level of tar in usual plasma gasification processes [96].

Although it is reported that thermal gasification is also used in processes like the decomposition of municipal waste [96], it is in fact an example of cold plasma pyrolysis, a thermal process operated at around 600 °C that decomposes plastic waste with the help of biological catalysts [91,233]. At this range of temperatures, this plasma process is regarded as a nonthermal plasma process.

While admittedly the energy required for this process is significantly lower than if one were to use thermal plasmas, the same shortcomings regarding economic viability in terms of the two models still apply. This is to say, it still corresponds to the recovery step in the waste management hierarchy model in Figure 1. There is also an additional shortcoming regarding the efficiency of gasification; since gasification is a purely thermal process, the low temperature of the nonthermal plasma is not advantageous to the speed of this procedure. Hence, gas production using nonthermal plasmas is less efficient than with thermal plasmas.

It is important to understand that plasma gasification is favourable if syngas or hydrogen are desirable products. However, some processes may require the use of syngas as a raw material in an organic compound. Since plasma reforming allows for the direct formation of favourable compounds, this is the more appropriate process for such instances.

While plasma gasification produces a syngas, the corresponding main product produced in plasma liquefaction is liquid fuel. It is evident that this process, much like plasma gasification, also corresponds to the recovery process in Figure 1 and is therefore also a tertiary recovery process. The production of liquids is not possible using thermal plasmas, since the temperatures achieved by thermal plasmas are too high for the target product fuel to remain as a liquid that can still be further decomposed.

Plasma liquefaction using nonthermal plasma is a process by which solid biomass is transformed into a liquid form, with the help of catalysts and solvents. This is an attractive process, since producing liquid forms of fuels is advantageous [234]. Note the two following reasons. Firstly, in applications other than in fuel, there is a preference to keep biomass in a form where their molecules are decently large: this usually corresponds to a liquid form. Secondly, transportation becomes easier when biomass is in a liquid form; the transport of gaseous fuels requires containers that can sustain high gas pressures.

### 5.2. Wind Turbine Blade Recycling

There are two major challenges in the recycling of wind turbine blades. The first challenge is that wind turbine blades have a complicated structure, and the separation of components can prove to be quite difficult [235]; therefore, primary recycling of these blades is challenging. The second challenge is that wind turbine blades are partially made from fibre-reinforced polymers (FRP), typically with high contents of inexpensive glass fibres.

FRPs are widely used in aerospace and aeronautics [236,237], construction [238,239,240], transportation [241,242], and of course, wind energy, among many other areas. This is due to their light weight, environmental friendliness, mouldability, resistance to corrosion, and general mechanical properties [242]. FRPs are mainly composed of two materials: 1. fibres that are made of glass, carbon, or natural fibre [243] among others and 2. polymer matrices, which are either thermoplastics or thermosets [244]. The fact that there are various combinations for FRPs provides different challenges in their decomposition and separation. Recycling by way of heating proves to be particularly difficult when thermosets are present in the FRP, as thermosets remain hard when exposed to heat [245]. In wind turbine blades, thermosets are generally used.

There are two main ways to separate glass fibres: first, by retaining the original shape of the fibre, e.g., recycling glass fibres by using nonthermal plasma, or second, by melting it at a high temperature [244]. In the former case, the physical properties of the fibre are severely degraded, while the latter may only be performed at the laboratory level [241]. Additionally, the fact that glass fibre is not so expensive in the first place decreases the merit of melting it, especially considering the cost associated with the recycling process. The volume fraction of polymers in wind turbine blades is not very large. Although polymer matrices in wind turbine blades are much more valuable for recycling than glass fibres, this means that the plasma gasification of wind turbine blades produces significant amounts of degraded glass while also producing a limited amount of syngas, even in comparison to the regular plasma gasification process, which is unfavourable. Therefore, the wind energy community considers that it is cheapest to directly dispose of FRPs in landfills, and this is what is done in the majority of cases [246,247,248,249]. In this respect, it may be considered that wind energy is not an environmentally friendly green technology. Admittedly, FRPs are also used in electronic parts including circuit boards, whose wastes are generally handled by thermal plasma as discussed before. The important difference when recycling electronic wastes is that it primarily aims at recycling valuable metal components.

From this, it is not surprising to see that plasmas are not commonly used to recycle FRPs in wind turbine blades. There have been attempts to upcycle the carbon fibre in FRPs via pyrolysis, but the problem is that the upcycled carbon fibre is still degraded in comparison to virgin carbon fibres. Even though the use of thermal plasma somewhat mitigates the amount of graphitization that is usually seen [250], the previous issues regarding heat degradation still stand. However, the recovery of carbon fibres from carbon FRPs via chemical swelling and plasma treatment has been performed with success; compared to the original fibres, the recycled carbon fibres had 93.55% tensile strength and 118.76% interfacial shear strength [251].

Recycling entire wind turbine blades has also been attempted [252,253], and while researchers seem to be hopeful despite the degraded recycled products, the fact that most wind turbine waste is disposed of in landfills suggests that research in this field has proved to be unsuccessful so far.

### 5.3. Recycling Lithium-Ion Batteries

Lithium-ion batteries (LIBs) are referred to as secondary batteries, and are widely used in cars, mobile phones, and the like.

Plasmas are commonly used in the production processes of LIBs [6,254,255,256,257,258,259,260,261,262,263,264,265,266,267,268,269,270,271,272,273,274,275,276,277]. Both thermal plasmas [278,279,280] and nonthermal plasmas [281,282,283,284,285] are used for these production processes. The recyclability of LIBs is limited, and there are also many challenges that such a process would entail, which include the variety of battery designs and the limitations of current recycling technologies [286,287]. There are many expensive components in a LIB that are preferably recovered rather than recycled, which complicates the recycling process further [286]. Therefore, components are selectively recycled, and for LIBs, this is the case for metal components.

Broadly speaking, there are two different methods of recycling LIBs:

1. A heat-based smelting process (pyrometallurgy);
2. A liquid-based leaching process (hydrometallurgy).

Of these, smelting is a thermal process for which thermal plasmas are often used [288]. However, nonthermal plasmas have also been used to recycle LIBs, albeit very briefly: for example, to heat and combust the byproduct gas in an LIB recycling process [287], and the used graphite anode in the LIB has been shown to be fully functional after reactivation using a plasma jet [255]. According to the corresponding patent, the temperature of the plasma torch is 1150 °C (ca. 900 K) [289]. Such a temperature can be easily achieved using a quenched gliding arc. So, in this review, it is assumed to be a nonthermal plasma. This process can be broadly considered as a part of recycling techniques, as properties of substances or materials under the subjects are somehow recovered or improved so that they are ready to use and or reuse. On a tangent, it is very important to consider that techniques which have previously been considered out of the scope of recycling may potentially be applied in relevant recycling processes.

The latter process would be an example of reuse, as in Figure 1 and Figure 2.

### 5.4. Paper Recycling

De-inking used paper is a useful and environmentally conscious process in recycling [290,291] and may be considered a tertiary recycling process that corresponds to the recovery and recycling processes in Figure 1 and Figure 2. While biological catalysts like cutinases are commonly used [290,291], nonthermal plasmas, like atmospheric-pressure helium DBD plasmas, are also employed to speed up these catalytic reactions in recycling [292]. One may use MW low-pressure plasma to increase the hydrophilicity of the paper and decrease its disintegration time. This treatment decreases the energy consumption of the de-inking process [292,293].

In publications that discuss de-inking, references are sometimes made to “plasma jets”. This is synonymous to a cold plasma torch, or a nonthermal plasma torch. In recent years, it has been discovered that cold plasma torches that use helium are more efficient at increasing the hydrophilicity of polymethyl methacrylate (PMMA) [294] than those that use argon. This increase in wettability was found through surface characterization techniques, using the contact angle measurements of PMMA with water [294]. When surface modification plays an important factor in finding a solution in recycling processes, the concepts of surface energy and surface tension, as well as simple surface characterisation techniques such as contact angle measurements, are considered to be important [295,296,297,298,299,300,301,302,303,304,305,306].

These examples indirectly assist recycling processes. Enthusiastically considering these indirect processes as plasma recycling techniques may lead to novel applications.

## 6. A Critique of Applying Plasmas in Recycling Processes

In general, thermal plasmas are suitable for thermal processes, while nonthermal plasmas better suit applications in surface processes and gas-phase reactions. Since each advantage is distinctively different, one possible perspective to synergistically combine the processes of thermal and nonthermal plasmas in recycling can be to employ a thermal plasma to generate gases, which are afterwards treated by a nonthermal plasma. Alternatively, after inorganic solids are processed by a thermal plasma, their surfaces may be modified by a nonthermal plasma.

Both thermal and nonthermal plasma processes are known to be environmentally friendly, dry processes that do not use solvents. However, one cannot neglect the expenses induced by employing them into any specific application. The investment cost including a plasma generator and a chamber usually dominates the expense of the process. It means that if the plasma system used is not efficiently used for mass production, the investment cost may become high.

Another important issue is the use of electricity within the running cost. Plasmas used for materials processing are electric gas discharges, which inevitably consume a certain amount of electric energy. If the mass consumption of electrical energy is an issue, the use of plasma should be avoided.

It is often the case that costs are the major driving force for selecting a process, and not the effectiveness of the technologies themselves. Since this is also the case for recycling processes, it is difficult to foresee future trends. For example, owing to recent serious developments in the generation and storage of electricity, as well as technological advancements, the above concerns regarding expenses may become minor issues.

From the viewpoints of the recycling classifications discussed in Section 2, it is desirable not to destroy waste down to a molecular level, but to preserve bulk properties. In this sense, nonthermal plasmas will play a more important role in solutions of recycling. However, in order for nonthermal plasmas to play more important roles in recycling, an overall change is required with respect to the design, manufacture, and suitability of the treatment to a given process.

## 7. Conclusions

When thermal plasmas are used for waste management, these are generated in plasma torches in all cases excluding spark plasma sintering. While the terminology differs from publication to publication, it is seen that the processes are essentially all the same. It has also been noted that when a large quantity of waste is thermally processed, this is most efficiently achieved by using a thermal plasma; this also helps achieve the maximum temperature. This is because the electrical impedance of thermal plasmas is low, so a high current can be generated from a low voltage. This leads to a high-power process, in which a high-temperature environment can easily be achieved from the Joule heat. With regards to DC, RF, and MW plasmas, if the contamination from the electrodes melting is not a significant issue, DC plasmas are the most efficient for large-scale processes.

The shortcomings of thermal plasmas are that a high power is needed to achieve a high temperature, and that they need an environment or a container resistant to high temperature. It is advisable to consider these limitations. Additionally, the residue waste after gasification will also be exposed to high temperatures; for cases in which this is not appropriate, it is important to select a process that does not reach very high temperatures.

The number of attempts that use nonthermal plasmas in recycling processes are much less than for thermal plasmas. This is due to the fact that nonthermal plasmas are unsuited for the high-speed processing of bulk solids or liquids. However, considering their selectivity, gas-phase reactions, and their affinity for surface reactions with solids and liquids, there are applications that are able to utilise these advantages.

Alternatively, one may consider nonthermal plasmas in processes that assist the main recycling process. In these cases, nonthermal plasmas may be found to be increasingly more useful. In reality, nonthermal plasmas are already used in recycling processes, even if they are not explicitly defined as such. In order to improve recycling processes in the future, this may be something worth considering.

## Figures and Tables

**Table 1:** Typical plasma parameters of plasma processes in air (values are rounded).

	Type of Plasma	Max. Temp. (K)		Electron Density (cm^−3^)	Applications	Refs.
		Electron	Gas			
Thermal	Plasma torch (DC, RF, MW)	10^4^	10^4^	10^15^	Gasification Metal recovery	[19]
	Spark plasma	10^4^	10^4^	10^15^–10^18^	Sintering	[20,21]
Nonthermal	Corona discharge	10^5^	10^2^–10^3^	10^8^	Surface modification Plasma reforming	[20,22,23]
	Dielectric barrier discharge	10^4^–10^5^		10^14^–10^15^		[24]
	Cold plasma torch	10^4^		10^14^		[25]
	Gliding arc	10^4^	2 × 10^3^	10^15^		[26,27]

**Table 2:** Comparison of plasma torches used for recycling.

	Electrode	Typical Max. Power Reported [kW]	Advantage	Disadvantage
DC plasma torch	Needed	118.8 [83]	High power applicable	Contamination by electrode erosion
RF plasma torch	No need	75 [84]	Electrodeless	Use of matching network
MW plasma torch	No need	10 [85,86]	Electrodeless, arguably energy efficient	Use of ignition system

## Data Availability

Data sharing is not applicable.

## Abbreviations

The following abbreviations are used in this manuscript:

MDPI	Multidisciplinary Digital Publishing Institute
DC	Direct current
AC	Alternating current
RF	Radiofrequency
MW	Microwave
ICP	Inductively coupled plasma
CCP	Capacitively coupled plasma
MSW	Municipal solid waste
ELM	Enhanced landfill mining
DBD	Dielectric barrier discharge
LIB	Lithium-ion battery
PMMA	Polymethyl methacrylate
FRP	Fibre-reinforced polymer
